# A multicenter, open-label, randomized, proof-of-concept phase II clinical trial to assess the efficacy and safety of icatibant in patients infected with SARS-CoV-2 (COVID-19) and admitted to hospital units without invasive mechanical ventilation: study protocol (ICAT-COVID)

**DOI:** 10.1186/s13063-022-06219-7

**Published:** 2022-04-12

**Authors:** Pierre Malchair, Aurema Otero, Jordi Giol, Xavier Solanich, Thiago Carnaval, Alonso Fernández-Nistal, Ana Sánchez-Gabriel, Carmen Montoto, Ramon Lleonart, Sebastián Videla, on behalf of the ICAT-COVID team, Arnau Antoli, Marta Benjumeda, Tania Bernal, Laura Calatayud, Xavier Corbella, Anna Ferrer, Vanesa Garcia, Mercè Gasa, Carlota Gudiol, Pilar Hereu, Javier Jacob, Hector Jofre, Ferran Llopis, Leire Matellan, Natalia Pallarés, Raul Rigo, Gemma Rocamora, Freddy Rodríguez, Alexander Rombauts, José Carlos Ruibal, Joan Sabater, Carmen Serrano, Ana Suárez-Lledó, Cristian Tebé, Jesús Villoria, Alvaro Zarauza

**Affiliations:** 1 Emergency Department, Bellvitge University Hospital, L’Hospitalet de Llobregat, Barcelona, Spain; 2 Clinical Research Support Unit (HUB-IDIBELL: Bellvitge University Hospital & Bellvitge Biomedical Research Institute), Bellvitge University Hospital, L’Hospitalet de Llobregat, Barcelona, Spain; 3 Internal Medicine Department, Bellvitge University Hospital, L’Hospitalet de Llobregat, Barcelona, Spain; 4 Clinical Pharmacology Department, Bellvitge University Hospital, L’Hospitalet de Llobregat, Barcelona, Spain; 5 Medical Department, Takeda Farmacéutica España S.A., Madrid, Spain; 6 Allergology Section, Internal Medicine Department, Bellvitge University Hospital, L’Hospitalet de Llobregat, Barcelona, Spain; 7 Pharmacology Unit, Department of Pathology and Experimental Therapeutics, School of Medicine and Health Sciences, IDIBELL, University of Barcelona, L’Hospitalet de Llobregat, Barcelona, Spain

**Keywords:** SARS, ACE2, ARDS, Bradykinin, SARS-CoV-2, COVID-19, Icatibant

## Abstract

**Background:**

COVID-19 has quickly become a global pandemic with a substantial number of deaths and is a considerable burden for healthcare systems worldwide. Although most cases are paucisymptomatic and limited to the viral infection-related symptoms, some patients evolve to a second phase, with an impaired inflammatory response (cytokine storm) that may lead to acute respiratory distress syndrome and death. This is thought to be caused by increased bradykinin synthesis.

**Methods:**

ICAT-COVID is a multicenter, randomized, open-label, proof-of-concept phase II clinical trial assessing the clinical efficacy and safety of adding icatibant to the standard of care in patients hospitalized with COVID-19 without invasive mechanical ventilation. Patients hospitalized with a confirmed COVID-19 pneumonia diagnosis (RT-PCR or antigen test ≤ 10 days prior to randomization, and radiographic evidence of pulmonary infiltrates), rated “4” or “5” on the WHO’s clinical status scale, are eligible. Patients will be randomized on a 1:1 ratio to either standard of care-plus-icatibant (experimental group) or to standard of care alone (control group). The experimental group will receive 30 mg of icatibant subcutaneously 3 times a day for 3 days (for a total of 9 doses). The expected sample size is 120 patients (60 per group) from 2 sites in Spain. Primary outcomes are the efficacy and safety of Icatibant. The main efficacy outcome is the number of patients reaching grades “2” or “1” on the WHO scale within 10 days of starting treatment. Secondary outcomes include “long-term efficacy”: number of patients discharged who do not present COVID-19-related relapse or comorbidity up until 28 days after discharge, and mortality.

**Discussion:**

Icatibant, a bradykinin type 2 receptor antagonist with proven effectiveness and safety against hereditary angioedema attacks, may be beneficial for COVID-19 patients by inhibiting bradykinin’s action on endothelial cells and by inhibiting the SARS-CoV-2 M protease. Our working hypothesis is that treatment with standard of care-plus-icatibant is effective and safe to treat patients infected with SARS-CoV-2 admitted to hospital for pneumonia without invasive mechanical ventilation.

**Trial registration:**

EudraCT 2020-002166-13. ClinicalTrials.gov NCT04978051

**Supplementary Information:**

The online version contains supplementary material available at 10.1186/s13063-022-06219-7.

## Administrative information

**Table:** 

**Title {1}**	**A MULTICENTER, OPEN-LABEL, RANDOMIZED, PROOF-OF-CONCEPT PHASE II CLINICAL TRIAL TO ASSESS THE EFFICACY AND SAFETY OF ICATIBANT IN PATIENTS INFECTED WITH SARS-CoV-2 (COVID-19) AND ADMITTED TO HOSPITAL UNITS, WITHOUT INVASIVE MECHANICAL VENTILATION. STUDY PROTOCOL (ICAT-COVID)**
**Trial registration {2a and 2b}.**	EudraCT: 2020-002166-13 ClinicalTrials.gov: NCT04978051.
**Protocol version {3}**	Version 3.2, January 21^st^, 2021
**Funding {4}**	This colaborative research clinical trial will receive funding and the experimental drug product (Icatibant) from Takeda. Funding from Takeda lab will be for covering the expenses related to administrative procedures for clinical trial start-up, fees of the IRB and of the AEMPS (Spain’s regulatory authority), the Contract Research Organization engaged to carry out study monitoring, pharmacovigilance, e-CRF preparation, statistical analysis, and the final report. Therefore, feasibility of this clinical trial depends on this financial contribution. Takeda, the marketing authorization holder of Icatibant, will also contribute with the experimental study product, valued at over one million euros.
**Author details {5a}**	**Dr. Pierre Malchair:** Emergency Department, Bellvitge University Hospital **Dr. Aurema Otero:** Clinical Research Support Unit (HUB-IDIBELL), Bellvitge University Hospital (HUB) & Bellvitge Biomedical Research Institute (IDIBELL). **Dr. Jordi Giol:** Emergency Department, Bellvitge University Hospital **Dr. Xavier Solanich:** Internal Medicine Department, Bellvitge University Hospital **Dr. Thiago Carnaval:** Clinical Pharmacology Department, Bellvitge University Hospital **Dr. Alonso Fernández-Nistal:** Medical Department, Takeda Farmacéutica España S.A. , Spain **Ana Sánchez-Gabriel, MSc:** Medical Department, Takeda Farmacéutica España S.A. **Dr. Carmen Montoto**; Medical Department, Takeda Farmacéutica España S.A. **Dr. Ramon Lleonart:** Allergology Unit, Internal Medicine Department, Bellvitge University Hospital **Dr. Sebastián Videla:** Clinical Research Support Unit (HUB-IDIBELL); Clinical Pharmacology Department, Bellvitge University Hospital; Pharmacology Unit, Department of Pathology and Experimental Therapeutics, School of Medicine and Health Sciences, IDIBELL, University of Barcelona
**Name and contact information for the trial sponsor {5b}**	Sponsor: Bellvitge Biomedical Research Institute (IDIBELL). Contact person: Dr. Sebastián Videla, Head of Clinical Research Department, Bellvitge Biomedical Research Institute (IDIBELL) email: svidela@bellvitgehospital.cat
**Role of sponsor {5c}**	The sponsor is responsible for ensuring and maintaining the quality and control systems throughout the study. The sponsor will also manage the funding provided by Takeda, and ensure the principal investigator (or whoever he/she delegates to) of each participating site receives funding to pay for the expenses generated by study activities such as hiring personnel, material, etc.
**Composition of the coordinating center and trial steering committee {5d}**	The coordinating center and the steering committee of the trial will include the sponsor, represented by Dr. Sebastian Videla, a specialist in clinical pharmacology and Head of Clinical Research Department, Bellvitge Biomedical Research Institute (IDIBELL); the coordinator of this clinical trial and Head of the Emergency Department, Dr. Pierre Malchair, and the medical monitors Dr. Jordi Giol and Dr. Xavier Solanich, both specialists in internal medicine.

## Introduction

### Background and rationale {6a}

The disease caused by the novel coronavirus SARS-CoV-2 (COVID-19) has become a global pandemic [1]. Since the start of the pandemic in March 2020, until January 31, 2022, the World Health Organization (WHO) has confirmed 380,321,615 cases and 5,680,741 deaths worldwide [2]. For the medical community, this new disease represents a challenge in identifying effective therapeutic options for treatment and prevention.

Multiple randomized clinical trials on COVID-19 are currently ongoing, and a wide range of drugs are being administered in clinical practice to adequately address the two clinical phases that the disease appears to have in its most severe manifestation: a first phase with viral infection predominance and a second phase with an exacerbated inflammatory response predominance [3], which causes, in most cases, acute respiratory distress syndrome (ARDS) in adult patients.

Icatibant acetate (Firazyr®) is a synthetic peptide, similar in structure to bradykinin, that acts as a competitive antagonist at the bradykinin type 2 receptor (BKB2R) on the endothelial cells [4]. Icatibant is approved for the treatment of acute attacks of angioedema in patients diagnosed with hereditary angioedema (HAE) [5, 6]. Its long-term safety has been proven with data from the Icatibant Outcome Survey: IOS registry [7, 8]. Icatibant has also been shown to be effective against ACE inhibitor-induced angioedema in some case series, as well as in real-life clinical experience [9]. It is noteworthy that ACE inhibitors-induced angioedema has bradykinergic characteristics and does not respond to corticosteroids, antihistamines or epinephrine.

Since the contact system is activated in ARDS, there is an increased bradykinin synthesis that triggers a local pulmonary inflammatory response due to activation of the bradykinin receptors of endothelial cell [10]. This increases vascular permeability and recruitment and activation of inflammatory mediators, causing the pulmonary edema seen in the disease’s early stages [11]. Moreover, SARS-CoV-2 binding to the respiratory epithelial cells’ ACE2 receptor decreases its activity [12], increasing plasmatic levels of des-Arg9-bradykinin, although a Chinese cohort study did not find an increased risk of infection with SARS-CoV-2 in hypertensive patients treated with ACE inhibitors [13].

The rationale for the potential efficacy of icatibant in COVID-19 is based on the inhibition of the action of bradykinin on endothelial cells and on the inhibition of the SARS-CoV-2 M protease (a key enzyme in coronavirus replication). This last mechanism of action was identified on a theoretical computational model [13]. Our working hypothesis is that adding icatibant to standard of care (SoC) is effective and safe in patients with pneumonia caused by SARS-CoV-2 (COVID-19) without invasive mechanical ventilation and admitted to hospital units. The purpose of this project is to conduct a proof-of-concept, multicenter, controlled, randomized clinical trial to provide the first evidence of our working hypothesis.

### Explanation for the choice of comparators {6b}

In January 2021, SoC, based on respiratory support measures, fluid therapy, antipyretic treatment, postural measures, low-molecular-weight-heparins, corticosteroids (such as dexamethasone), and remdesivir (put in question), was the treatment of choice. With the appearance of new potential treatments during the study, we allow the use of every authorized drug as standard of care [14] in order to offer the best treatment to patients.

In this clinical trial, therefore, adding icatibant to SoC will be compared to SoC alone for hospitalized COVID-19 patients with pneumonia caused by SARS-CoV-2 (COVID-19) without invasive mechanical ventilation.

### Objectives {7}

#### Primary objective

The main objective of the ICAT-COVID study is to assess the efficacy and safety of icatibant 10 days after starting treatment (or at hospital discharge if this occurs before) in adult inpatients with SARS-CoV-2 pneumonia and without invasive mechanical ventilation.

#### Secondary objectives

The secondary objectives are as follows: (1) to study the time to achieve a sustained clinical response for 48 h, (2) to study the time to reach a sustained afebrile state for 48 h, (3) to study long-term efficacy 28 (± 3) days after hospital discharge, (4) to study the role of pharmacological treatments used in SoC in efficacy and safety, (5) to quantify hospital stay duration (in days), (6) to assess the influence of elapsed time from symptom onset (days with the disease) on treatment response, (7) to assess the incidence of COVID-19-related complications up to 28 (±3) days after hospital discharge, (8) to assess the incidence of SARS-CoV-2 pneumonia relapse from hospital discharge up until 28 (±3) days later, (9) to estimate the incidence of all-cause new emergency room visits from hospital discharge until 28 (±3) days later, (10) to estimate the incidence of mortality due to COVID-19 up to 28 (±3) days after hospital discharge, (11) to estimate the incidence of all-cause mortality through 28 (±3) days after hospital discharge, and (12) to estimate the incidence of adverse events (AEs) by severity up to 28 (±3) days after hospital discharge. To evaluate the safety of icatibant: incidence of adverse events by severity up to 28 (±3) days after hospital discharge.

### Trial design {8}

This study is a phase II, randomized, controlled, open-label, proof of concept, multicenter clinical trial with two parallel groups (experimental group: SoC-plus-Icatibant, and control group: SoC) to assess the efficacy and safety of icatibant in patients with COVID-19 pneumonia without invasive mechanical ventilation, and admitted to hospital units. The outcomes of the study protocol are based on the draft master protocol of the WHO for clinical trials that evaluate the efficacy and safety of investigational therapeutics for the treatment of COVID-19 in hospitalized patients (Table 1) [15].

**Table 1:** WHO 8-point ordinal scale for clinical status assessment

Patient status	Score
Uninfected: No clinical or virological evidence of infection	**0**
Ambulatory, with no limitation of activities.	**1**
Ambulatory, with limitation of activities.	**2**
Hospitalized, not requiring supplemental oxygen	**3**
Hospitalized, requiring low-flow supplemental oxygen (mask or nasal prongs)	**4**
Hospitalized, on non-invasive ventilation or high-flow oxygen devices.	**5**
Hospitalized, intubated and mechanical ventilation.	**6**
Hospitalized, on invasive mechanical ventilation and additional organ support: extracorporeal membrane oxygenation (ECMO), renal replacement therapy, pressors.	**7**
Death	**8**

Patients enrolled in the study will be randomly assigned at a 1:1 ratio to the experimental group and the control group. The experimental treatment will consist of SoC-plus-Icatibant (3 doses per day for 3 consecutive days (a total of 9 doses). Each dose will consist of 30-mg icatibant administered subcutaneously. The control treatment will consist of SoC only.

Since the beginning of the COVID-19 pandemic, SoC has been mostly supportive, considering the lack of evidence for highly effective therapies. So SoC could vary depending on the scientific evidence available during the study. In this clinical trial (ICAT-COVID), SoC could include respiratory support measures, fluid therapy, antipyretic treatment, postural measures, low-molecular-weight-heparins, corticosteroids such as dexamethasone, remdesivir, and other therapeutic options.

The diagnosis of SARS-CoV-2 pneumonia will be made in hospitalized patients presenting de novo radiographic infiltrates (either by plain chest radiography or computed tomography), and a confirmed SARS-CoV-2 infection as determined by RT-PCR or antigen test for SARS-CoV-2. Clinical status will be assessed using the WHO 8-point ordinal scale for clinical assessment (Table 1).

## Methods: participants, interventions, and outcomes

### Study settings {9}

ICAT-COVID will be carried out at 2 Spanish healthcare centers:

- Bellvitge University Hospital, L’Hospitalet de Llobregat, Spain
- Martorell Hospital, Martorell, Spain

### Eligibility criteria {10}

*Inclusion criteria:*

- Adult patients (≥ 18 years old), of both genders.
- SARS-CoV-2 infection confirmed by laboratory RT-PCR or antigen test ≤ 10 days prior to randomization.
- Hospitalized with a diagnosis of SARS-CoV-2 pneumonia.
- Radiographic evidence of pulmonary infiltrates of ≤ 48.
- Patients rated “4” or “5” on the WHO 8-point ordinal scale for clinical status assessment.
- PaO_2_/FiO_2_ < 380.
- Men and women of childbearing age who have heterosexual intercourse must agree to use safe contraceptive method(s).
- Subject or legally authorized representative must sign a written informed consent that states that he/she understands and agrees to comply with the planned study procedures.

*Exclusion criteria:*

- Imminent death (life expectancy ≤ 24 h)
- Known hypersensitivity or known adverse reactions to the study drug, its metabolites, or excipients of the formulation
- Invasive mechanical ventilation
- Participation in any other clinical trial
- ALT or AST blood levels > 5 × ULN (upper limit of normal)
- Creatinine clearance < 50 mL/min using the Cockcroft-Gault formula for participants ≥ 18 years old [13]
- Patients with a recent acute coronary syndrome (< 1 month)
- Patients with cerebrovascular accident background
- Women who are pregnant or breastfeeding

### Interventions

#### Intervention description {11a}

Takeda has provided the experimental study product: icatibant acetate (Firazyr®, Shire Pharmaceutical Ireland Limited. Block 2 & 3 Miesian Plaza, 50–58 Baggot Street Lower. Dublin 2, Ireland). The clinical research organization (Bioclever, Rambla Catalunya, 135, 3° 1ª, 08008-Barcelona) engaged for monitoring and pharmacovigilance was in charge of delivering the medication (icatibant) to the hospital pharmacies.

The e-CRF will provide the random code after the investigator introduces the required data. Subsequently, for the first dose, the investigator of each center will contact the person in charge at the hospital pharmacy. Icatibant will be prepared straight after randomization at the hospital pharmacy and the hospital pharmacist will provide it to the nurse in charge of administering it. Subsequent doses (from 2nd to 9th doses) will be scheduled according to usual nursing clinical practice procedure.

As abovementioned (explanation for the choice of comparators {6b} section), in January 2021, SoC was based on respiratory support measures, fluid therapy, antipyretic treatment, postural measures, low-molecular-weight-heparins, corticosteroids (such as dexamethasone), remdesivir, and other therapeutic options. The hospital pharmacy of each center will provide these drugs according to usual clinical practice procedure.

#### Criteria for discontinuing or modifying allocated interventions {11b}

Participants may voluntarily discontinue trial treatment and/or prematurely end their participation in the study for any reason, at any time. The investigator may also decide, at any time during the study, to temporarily interrupt or permanently discontinue the trial treatment if it is deemed that continuation would be detrimental to the participant or not in the best interests of the participant.

Similarly, the sponsor, Ethics Committee (EC), or authorized regulatory authority can decide to halt or prematurely discontinue the clinical trial if new information becomes available whereby the rights, safety, and well-being of trial participants can no longer be assured, when the integrity of the study has been compromised, or when the scientific value of the study has become obsolete and/or unjustifiable.

Circumstances requiring premature treatment interruption or discontinuation of the clinical trial include, but are not limited to, (1) safety concerns related to blood product or unacceptable intolerability (potentially life-threatening reaction during treatment), (2) trial participation while in breach of the eligibility criteria, and (3) pregnancy or the intention of becoming pregnant. In any such case of early trial termination and/or treatment interruption/discontinuation, the investigator will continue to closely monitor the participant’s condition and ensure adequate medical care and follow-up. Additionally, these patients will continue to be followed up for the primary outcome and their data will be included in intention-to-treat analyses.

#### Strategies to improve adherence to interventions {11c}

This study will be carried out on patients admitted to hospital units and treatment will be administered by healthcare professionals under the study team’s supervision. Detailed information on the administration of interventions is provided within the hospital’s electronic system (SILICON) and will be displayed once the nurses administer treatment. Most follow-up visits will be performed during hospital admission. The only visit to be performed on outpatients (visit 7) will be, a priori, a phone call visit, and patients would be requested to come to the hospital for a face-to-face visit only if considered necessary by the evaluating physician.

#### Relevant concomitant care permitted or prohibited during the trial {11d}

##### Concomitant treatment

According to the Summary of Product Characteristics (items 4.3. Contraindications; 4.4. Special warnings and precautions for use; and 4.5. Interaction with other medicinal products and other forms of interaction), icatibant could be administered concomitantly with any drug.

Based on our experience in patients like those proposed to be enrolled in this clinical trial: “patients with SARS-CoV-2 pneumonia with a short time since onset of pneumonia and who need to be admitted to hospital units to be treated with supplemental oxygen using non-invasive ventilation”, approximately 90% of these symptomatic patients with COVID-19 present a self-limited disease course within 7–10 days, and, therefore, are discharged from the hospital (time of the efficacy assessment).

On the other hand, icatibant has a short half-life (1.4 h; within 7–10 h after the last dose, the drug is cleared) and will be administered 3 times a day for 3 days. If the last dose is administered 64 h after the first dose (icatibant administrations at 0, 8, 16, 24, 32, 40, 48, 56, and 64 h), it is expected that at approximately 74 h (start of day four of the trial) icatibant would already be cleared.

One of the main risks for a patient hospitalized for pneumonia and treated with supplemental oxygen through non-invasive ventilation is superinfection (usually bacterial). If this happens during the study, antibiotics may be administered in the first days of the study and are not contraindicated during the use of icatibant.

As a result, the use of any drug will be permitted if considered necessary for clinical management. Any concomitant medication will be recorded in the clinical history (detailing the product, dose, route, days of administration and reason for treatment). All this information will be recorded in the e-CRF.

##### Rescue therapy

If the participant’s condition deteriorates, rescue therapy will be established according to the study protocol of each hospital. In general, these patients will receive the established SoC measures, consisting of respiratory support, fluid therapy, antipyretic treatment, postural measures, and low molecular weight heparins. It may also include anti-inflammatory treatment with corticosteroids, “anti-COVID antivirals” such as remdesivir, and other therapeutic options approved by the authorities.

##### Criteria for the discontinuation of the study treatment

As specified in the Summary of Product Characteristics, icatibant (according to the SmPC for Firazyr®) is contraindicated for hypersensitivity to the active ingredient or to any of its excipients.

### Outcomes {12}

#### Primary outcomes

Primary outcomes are the efficacy and safety of icatibant: number (percentage) of patients considered as responders. Responder is defined as a patient who reaches a clinical response [sustained 48-h grade 2, grade 1, or grade 0 classification on the WHO 8-point ordinal scale for clinical status assessment (Table 1)] *and* who has not presented any severe adverse events (grades “3”, “4,” or “5” of the Common Terminology Criteria for Adverse Events -CTCAE- (Table 2)) within 10 days after starting treatment.

**Table 2:** Common Terminology Criteria for Adverse Events (CTCAE) Version 4.0

Classification	Magnitude of the adverse event
**Grade 1**	Mild adverse event. Asymptomatic or mild clinical symptoms or just an observation; intervention not indicated.
**Grade 2**	Moderate adverse event. Minimal, local or non-invasive intervention indicated.
**Grade 3**	Serious or medically significant adverse event, but not immediately life-threatening. Hospitalization or hospitalization prolongation indicated; important limitation of self-care.
**Grade 4**	Adverse event with risk of mortality or disability.
**Grade 5**	Death associated with an adverse event.

#### Secondary outcomes

Clinical secondary outcomes are as follows: (1) “long-term efficacy”: number (percentage) of patients who reached a sustained 48-h grade 2, grade 1, or grade 0 classification on the WHO 8-point ordinal scale for clinical status assessment 10 days after starting treatment and within 28 days after hospital discharge; (2) time to achieve a clinical response: time (days) between visit 1 (treatment start visit) and the day of recording sustained 48-h grade 2, grade 1, or grade 0 classification on the WHO 8-point ordinal scale for clinical status assessment; (3) time to achieve an afebrile state: time (days) to reach a 48-h sustained afebrile state (i.e., body temperature ≤ 37.5 °C) without antipyretic medication; (4) time from symptom onset: time (days) between the first COVID-19-related symptom and visit 1 (treatment start visit); (5) number (percentage) of patients considered as responders based on National Early Warning Score 2 (NEWS 2)*. Responder is defined as a patient who reaches at least a sustained 24-h score ≤ 2; and (6) COVID-19-related relapse: number (and percentage) of patients discharged who presents a COVID-19-related relapse or comorbidity.

*Clinical severity assessment will be carried out using the NEWS 2: https://www.mdcalc.com/national-early-warning-score-news-2 [16]. This score, accessed by electronic devices, has demonstrated the ability to determine the degree of illness of a patient and prompts critical care intervention. The score is based on 7 clinical parameters (respiratory rate, oxygen saturation, any supplemental oxygen requirement, temperature, systolic blood pressure, heart rate, level of consciousness [Alert, Responsive to voice, Responsive to pain, Unresponsive]).

Safety secondary outcomes are as follows: (7) number (and percentage) of patients with any AEs; (8) number (and percentage) of AEs; (9) number (and percentage) of SAEs: according to their severity and relationship with treatment, and by determining the number of patients with a grade 3, grade 4, or grade 5 adverse event according to the CTCAE (Common Terminology Criteria for Adverse Events—see Table 2); (10) mortality for any cause: number (and percentage) of patients who died; (11) COVID-19-related mortality: number (and percentage) of patients who died due to COVID-19; (12) time until death: number of days from visit 1 until death; (13) COVID-19-related complications: number (and percentage) of patients who had any complications and number (and percentage) of complications up to 28 days after hospital discharge (visit 7); (14) number (and percentage) of patients who require intensive care; (15) number (and percentage) of patients requiring intensive care and invasive mechanical ventilation; (16) number (and percentage) of patients who require oxygen supplementation from hospital discharge up until visit 7; (17) number (and percentage) of patients diagnosed with another nosocomial infection; and (18) number (and percentage) of patients who requires hospital readmission within 28 (± 3) days from hospital discharge.

Safety assessment will be carried out from the treatment’s first dose up to 28 (±3) days after hospital discharge according to the CTCAE (all AEs, SAEs, and SUSARs will be reported in the e-CRF).

### Participant timeline {13}

See the participant timeline in Table 3.

**Table 3:** During the study, laboratory tests and other complementary tests will be performed according to the clinical criteria of the physician in charge. (1) According to medical criteria. (2) This visit will be conducted by phone call or face-to-face if the physician in charge deems necessary. (3) Before administering treatment. (4) Fever in hospitalized patients with SARS-CoV-2 pneumonia is usually measured on the forehead with an infrared thermometer every 6 h or on demand, if the patient’s status so requires. (5) Fever in patients hospitalized for SARS-CoV-2 pneumonia, generally, is taken on the forehead with an infrared thermometer every 6-8 hours or on demand if the patient’s status so requires. Respiratory parameters: O2 saturation and respiratory rate. (6) Includes complete cell blood count, D-dimer, sedimentation rate, AST, ALT, bilirubin, creatinine, creatinine clearance, LDH, PT, aPTT, fibrinogen, ferritin, HDL, LDL, triglycerides, troponin, creatine-kinase, PCR, complement factor C4, C1 inhibitor antigen, and C1 inhibitor functional activity

		Visit 1	Visit 2	Visit 3	Visit 4	Visit 5	Visit 6	Visit 7 ^**(2)**^
	Patient screening	Treatment start	Hospitalization			Efficacy assessment	Hospital discharge	Study end
**Days of treatment**		**Day 1**	**Day 2**	**Day 3 (last dose)**				
**Days since the beginning of treatment administration**		**Day 0**	**Day 1**	**Day 2**	**Day 3**	**Day 10 (±1)**		**Day 28 (±3) from hospital discharge**
**Obtaining informed consent**	✔							
**Eligibility criteria assessment**	✔							
**Demographic data**	✔							
**Acute illness information (SARS-CoV-2 pneumonia):**	✔					✔		
**▫ New-onset radiological infiltrates** ^**(4)**^								
**▫ RT-PCR or antigen testing: positive**								
**General examination [respiratory parameters, fever]** ^**(5)**^	✔	✔	✔	✔	✔	✔	✔	
**General laboratory tests** ^**(6)**^	✔	✔	✔	✔	✔^(1)^	✔	✔^(1)^	✔^(1)^
**12-lead EKG**	✔	✔	✔	✔	✔	✔	✔	✔
**▫ Patient Clinical Status Scale**	✔	✔	✔	✔	✔	✔	✔	✔
**▫ Clinical Severity Assessment Scale**								
**Treatment administration**		✔	✔	✔				
**Biomarker samples**		✔^(3)^	✔	✔		✔	✔	✔
**AE, SAE and SUSAR notification**	✔	✔	✔	✔	✔	✔	✔	✔
**Concomitant treatment recording**	✔	✔	✔	✔	✔	✔	✔	

### Study procedure

Patients will be screened at the emergency department of each hospital or on the hospitalization ward on day 1 of admission. Patients enrolled on the study will be followed up until 28 days (1 month) from hospital discharge.

At the *baseline visit* (day 0, patient screening), a complete evaluation to confirm that the patient meets all the inclusion criteria and none of the exclusion criteria will be carried out. Every patient will be informed about the study and will be given the information sheet and informed consent to sign prior to enrollment in the clinical trial. Demographic parameters, medical history, information on concomitant medication, and the presence of adverse events will be recorded.

At *visit 1* (treatment start visit), a new clinical assessment will be performed. Patients will be re-evaluated to confirm (double-check) that they meet all the inclusion criteria and none of the exclusion criteria. Patients will be randomized and assigned to one of the treatment arms. Treatment will then be started. One dose of icatibant will be given every 8 h for 3 days, for a total of 9 doses, for patients assigned to the experimental group, along with SoC.

Baseline visit and visit 1 may overlap. If this occurs, the investigator will collect all the information from both visits in the electronic case report form (e-CRF).

During hospital admission (number of days may vary by length of hospital admission), all outcomes related to the patient’s clinical status and clinical severity assessment criteria will be collected daily from the medical records. All medication received by patients, as well as fluid therapy and the presence of adverse events, will also be recorded.

The following information will be retrieved until day 4 of hospitalization: general examination, vital signs (including SpO_2_), clinical data for study outcomes and adverse events assessment, 12-lead EKG, and laboratory analysis (including renal function, hepatic enzymes, and parameters suggestive of ischemia—creatine kinase, troponin). Serious adverse events (SAEs) and all any AEs will be collected, even if they are not part of the study’s endpoints or related with the investigational drug.

*Visits 2* and *3* will correspond to safety assessment during hospital admission. Visit 2 and visit 3 will take place, respectively, on day 2 (24 h after treatment start, before the 4th dose of icatibant) and on day 3 (48 h after treatment start, before the 7th dose of icatibant). *Visit 4* will take place 72 h after treatment start, end of treatment’ visit (after all doses are administered). Hospital discharge may occur after completing visit 4, if the patient is clinically stable (i.e., no fever for 48 consecutive hours without antipyretic medication, PaO_2_/FiO_2_ > 380 or O_2_ saturation > 94%, respiratory rate ≤ 24 bpm, absence of decompensation of any comorbidity).

*Visit 5* [10 (± 1) days after treatment start] will be an efficacy assessment visit. A clinical assessment will be performed and the patient’s clinical status and clinical stability criteria^#^, as well as the medication used and the presence of adverse events, will be reevaluated. If hospital discharge visit overlaps with the day 10 visit, the investigator will only complete the day 10 visit in the e-CRF, clarifying that it is also the hospital discharge visit. The patient will be sent home and will be informed of the confinement regimen measures to be followed at home (or similar, e.g., medicalized hotel), if necessary.

A patient classified as a treatment failure (i.e., non-responder—any patients who do not meet the criteria to be considered a responder) will have their participation in this study terminated. Afterwards, their condition will be treated at the discretion of the physician in charge of their particular case.

^#^Clinical stability (defined as (1) body temperature ≤ 37.5 °C for 48 h without any antipyretic medication, (2) PO_2_/FiO_2_ > 380 or oxygen saturation > 94%, (3) respiratory rate ≤ 24 rpm, and (4) the absence of any kind of comorbidity decompensation) will also be assessed and the time to achieve it will be calculated starting from the time of the baseline visit.

If the patient is discharged before the 10th day since treatment start, the investigator must complete the day 10 visit in the e-CRF for the efficacy assessment on the same day as hospital discharge.

If the patient is discharged after the 10th day since treatment start, the date of hospital discharge will be recorded (*visit 6*). The following data will also be collected at the time of hospital discharge: the patient’s clinical status and clinical stability, the number of days on invasive mechanical ventilation (if required), the number of days on antiviral treatment (if administered), AEs, and SAEs.

The end of the study visit will be performed 28 days after hospital discharge. The patient will be informed that they will be called by phone on the 28th day after hospital discharge (*visit 7*) to assess the course of the disease. Patients will also be informed that they might be requested to report for a face-to-face appointment at the hospital, if considered necessary by the physician performing the phone interview. The aim of this visit is to collate potential relapses, readmissions, or new visits to the emergency room; information on complications related to COVID-19; number of deaths from any cause and cause of death (from hospital admission and up to 28 days after hospital discharge); and presence of AEs will also be collected.

### Sample size {14}

No formal sample size estimation was performed. Until now, no clinical trial has been published on efficacy and safety of icatibant in patients infected with SARS-CoV-2. Therefore, previous data on the efficacy of icatibant in patients with SARS-CoV-2 pneumonia to be able to estimate a sample size is not available. Furthermore, the statistical hypothesis of this proof-of-concept trial is not to demonstrate the superiority of “SoC-plus-Icatibant” versus “SoC” in efficacy. Our intention is to include a total of 120 patients (60 in the experimental treatment group and 60 in the control treatment group). The results of this clinical trial will provide information that will allow us to calculate the sample size required for the superiority hypothesis regarding the primary endpoint of the trial, which could be the objective of a future clinical trial.

### Recruitment {15}

The target population of this clinical trial are hospitalized patients diagnosed with SARS-CoV-2 pneumonia who are not on invasive mechanical ventilation (i.e., grades 4 and 5 of the WHO 8-point clinical status assessment ordinal scale—see Table 1). This type of patient usually arrives at the emergency department of each hospital. The investigators of each center will recruit the study subjects prospectively and include them in the study for subsequent randomization at the emergency department if they meet the inclusion criteria and if there is no reason for exclusion (see the “Eligibility criteria {10}” section). The hospitals involved in this clinical trial will avoid participating in other clinical trials that may compete for the same type of patients.

## Assignment of interventions: allocation

### Sequence generation {16a}, concealment mechanism {16b}, and implementation {16c}

Participants will be block randomized using an Internet-based, concealed computer-generated random allocation sequence. Random blocks will be of size 6 and stratified by hospital. The randomized sequence allocation will be stored in the Biostatistics Unit at Biomedical Research Institute of Bellvitge (IDIBELL) and will not be available to any member of the research team. The randomization process will be performed by the investigator using the e-CRF (REDCAP). The randomization list will be computer-generated in blocks of four and stratified for each site. Patients will be assigned to the study groups at the time of enrollment in the study.

## Assignment of interventions: blinding

### Who will be blinded? {17a}

This is an open-label clinical trial; the study medication will not be blinded.

### Procedure for unblinding if needed {17b}

This does not apply because it is an open-label study.

## Data collection and management

### Plans for assessment and collection of outcomes {18a}

Data of interest (see primary and secondary outcomes) will be collected by the study participants’ physicians, who are familiar with this protocol. It will be collected from the anamnesis and from physical examination, which will be recorded in the patients’ digital clinical history, as well as the complementary test information. In both groups, patients will be monitored daily during the first 4 days of hospitalization and the patient’s clinical status and clinical response to treatment will be assessed.

The sponsor will oversee the maintenance of quality control systems while this study is being conducted, using standard operating procedures to do so. Data entry personnel will be given access to the participants’ digital clinical history, so that they can complete the e-CRF.

All trial-related parties accept direct access to the data source, and to the documents and reports related to the study, to be monitored and audited by the sponsor, and for inspection by the regulatory authorities. Source documents of the study must be traceable.

### Plans to promote participant retention and complete follow-up {18b}

All control visits except the last ONE will be carried out during hospitalization. This last control follow-up visit after hospital discharge will primarily be a phone call interview (carried out by one of the study participant’s physicians). At the evaluating physician’s discretion, a face-to-face medical follow-up visit may be indicated. We expect this straightforward follow-up procedure to encourage participant retention and allow the follow-up process to be properly carried out.

Protocol deviations will be documented and explained in detail by the investigators, and the sponsor will be informed, via the study monitor. In the event of a serious protocol violation, the investigator must inform the sponsor immediately. The monitoring team will record all protocol deviations. The sponsor will review all protocol deviations and assess whether any represent a serious breach according to good clinical practice guidelines. The sponsor will inform the institutional review board (IRB) of any protocol deviations that could impact patient safety and data integrity.

### Data management {19}

An electronic case report form (e-CRF) based on REDCap platform (Research Electronic Data Capture software, REDCap Consortium) will be created ad hoc for this study in coordination with the Biostatistics Unit of the IDIBELL (UBiDi). It does not collect data that allows patient identification. Stratification by healthcare center will be implemented when programming the e-CRF. When the computer system assigns the treatment group, therefore, stratification by healthcare center will have already been taken into account.

Data regarding random assignment (date and time), demographic information, clinical history, important clinical complications, clinical data on the acute condition, etiological agent, and hematological and biochemical analysis will be extensively collected, as well as the data regarding the doses of the study treatment administered and of any other treatment.

## Statistical methods

### Statistical methods for primary and secondary outcomes {20a}

All data collected in the study will be summarized using appropriate statistical methods. These summaries will be stratified by study group.

First, a descriptive analysis of the study variables will be performed. Continuous variables will be reported as mean and standard deviation (SD) or as median and range, and categorical variables will be described as absolute frequencies and percentages.

To assess the efficacy of icatibant in adult patients hospitalized for COVID-19, 10 days after starting the treatment, the difference between both groups’ response rate to treatment will be calculated [relative risk [RR] and its 95% of confidence interval (95%CI)].

The primary outcome analysis will be per protocol (PP). If a relevant proportion of subjects (> 10%) is found to present relevant protocol deviations (e.g., failure to meet screening criteria, visit calendar, or technical aspects of the study treatment), a PP analysis group would be defined (from which subjects with such deviation would be excluded). If this happens, all exploratory efficacy analyses would be performed once more for this PP group as a sensitivity test. Protocol deviations will be settled prior to starting data analysis (at the statistical analysis plan meeting). At that time, a decision will be made on whether the PP analysis group will be defined. The primary outcome will also be analyzed in the intention-to-treat population. This analysis should include all the subjects from the safety analyses set for whom an efficacy assessment is also available 10 days after starting the study treatment or throughout the study. Efficacy analyses will be performed on this group.

For the assessment of secondary outcomes, the unadjusted and adjusted estimates of the effect size and their corresponding 95% CI will be calculated using linear regression, logistic regression, or Cox’s proportional hazards regression.

Adverse events recorded during the study will be coded according to the latest available version of the MedDRA dictionary and described using absolute and relative frequencies by study group, according to severity and relation to treatment. We plan to perform three safety visits during hospitalization (visit 2, visit 3, and visit 4) aimed at collecting possible adverse events, on the first 10 patients recruited. This will allow for safety to be assessed before administering dose 4 and dose 7 of icatibant. If the safety evaluation of the first 10 patients is favorable (i.e., no significant adverse events are observed), the patient recruitment process will continue.

Lastly, the statistician performing the data analysis will be blinded regarding the treatments received by patients (experimental group: SoC-plus-icatibant vs. control group: SoC). R version 3.6.2 or higher for Windows (R Foundation for Statistical Computing) will be used for data processing and analysis.

### Methods for additional analyses (e.g., subgroup analyses) {20b}

A multivariate regression model will be constructed and adjusted for potentially significant confounding factors such as age, gender, clinical complications, and baseline symptoms.

### Methods in analysis to handle protocol non-adherence and any statistical methods to handle missing data {20c}

In the case of missing data, imputation will be made taking into account that the treatment effects’ estimator is not biased and that an increase in type I error has been avoided. Since the disease under study tends to present a progressively worsening natural course, it may be complicated to apply imputation techniques that respect these two conditions. However, considering that the aforementioned hypothesis contrast ensures the clinical trial’s internal sensitivity as an improvement in the experimental group is expected, the last observation carried forward (LOCF) technique seems to be a conservative approach in this regard.

## Oversight and monitoring

### Composition of the data monitoring committee, its role, and reporting structure {21a}

A data safety and monitoring board (DSMB) will be created ad hoc of a physician or pharmacist with expertise in pharmacovigilance, and of a physician with COVID-19 expertise, both external to this protocol, and by a physician from the sponsor. The aim of this DSMB is to evaluate the safety of this clinical trial and to decide whether or not to go ahead with patients’ inclusion. Two meetings are planned. The first one with the first 10 patients enrolled and the second one with the first 42 patients included. Patients’ inclusion will be stopped after the inclusion of patient number 10 until the DSMB has issued a decision regarding the continuation of the study.

Study monitoring will be carried out by a contract research organization (CRO) hired for this purpose and separate to the trial team. The sponsor and investigator/participating site will allow direct access to the trial data and corresponding data source, and to any other trial-related documents or materials to verify the accuracy and completeness of the data collected.

### Interim analyses {21b}

An interim analysis will be performed when 35% of the sample size (42 patients) has been enrolled and randomized and has completed the 10-day follow-up period or has been discharged from hospital, if this occurs before 10 days. The aim of this interim analysis is to assess the efficacy and the futility for evaluating the possible termination of the study (if futility issues are identified).

### Adverse event reporting and harms {22}

Adverse events recorded during the study will be coded according to the latest available version of the MedDRA dictionary and will be described using absolute and relative frequencies by study group, according to severity and its causal relation with treatment.

We intend to perform three safety visits during the hospitalization period (visit 2, visit 3, and visit 4), aiming to collect possible adverse events, in the first 10 patients recruited. This will allow a safety assessment before administering the 4th and the 7th doses of icatibant. If the safety evaluation of the first 10 patients is favorable (i.e., no notable adverse events are observed), the recruitment process will continue.

Serious adverse events will be described by study group and the 95%CI of the efficacy difference between both groups will be calculated. Peto’s method will be used by setting the stopping boundaries at *p* < 0.01. The study will be terminated if futility or safety issues are identified.

### Frequency and plans for auditing trial conduct {23}

The investigator shall allow direct access to trial data and documents for monitoring, audits, and/or inspections by authorized entities such as, but not limited to, the sponsor or its designees and competent regulatory or health authorities. As such, e-CRFs, source records, and other trial-related documentation (e.g., the trial master file, pharmacy records) must be kept current, complete, and accurate at all times. The auditors will be separate to the clinical trial and its conduct.

### Ethics approval and consent to participate {24}

The IRB of Bellvitge University Hospital acted as the trial’s coordinating ethics committee. The study protocol, version 3.2, was approved on March 5, 2021.

### Plans for communicating important protocol amendments to relevant parties (e.g., trial participants, ethical committees) {25}

As per good clinical practice, trial participants will be informed of any significant changes during the clinical trial. Major protocol changes will be submitted for IRB approval and minor outcomes will be informed to the IRB.

### Who will take informed consent? (26a)

Potential participants will be screened on the emergency ward or upon arrival at the COVID-ward. One of the physicians involved in the study will provide the first assessment of whether the patient is interested in participating in the study. If he/she is interested in participating, the investigators of the ICAT-COVID study team will double-check eligibility criteria and contact the patient to provide more information and obtain signed written informed consent.

The informed consent form includes a short and comprehensible summary of the rationale of the study, its design, and the drug. This is followed by a more detailed form, explaining all study-related procedures, clinical data collection (for instance clinical scores and vital signs), bio-sample collection (for instance blood samples and nasal swabs), and the potential risks (potential adverse events) and benefits (potential individual positive effects of the intervention, contribution to knowledge production). Also, data management and ethical approval are described, as well as the insurance policy. The investigator will also verbally explain this consent form and prior to asking the patient to sign the consent form they will be available to answer any questions related to the study that may occur. A copy of the informed consent form is attached to this manuscript as Supplementary Material: IC Form.

## Additional consent provisions for collection and use of participant data and biological specimens {26b}

Not applicable.

### Confidentiality {27}

The results from this clinical trial are confidential and may not be transferred to third parties in any form or manner without written permission from the sponsor. All individuals involved in the clinical trial are bound to this confidentiality clause in line with REGULATION (EU) 2016/679 OF THE EUROPEAN PARLIAMENT AND OF THE COUNCIL of April 27, 2016, on the protection of natural persons with regard to the processing of personal data and on the free movement of such data, as well as all other valid and applicable laws and regulations, such as “Ley Orgánica 3/2018, de 5 de diciembre, de Protección de Datos Personales y garantía de los derechos digitales” [the Spanish law on protection of personal data and guarantee of digital rights]. Therefore, patient data will be pseudonymized.

While obtaining a signature for the written informed consent, the investigator will request written permission from the patient to directly access their data. Once this permission is granted, the patient’s data may be examined, analyzed, verified, and reproduced for the evaluation of the clinical trial.

Data will be anonymized, so that the corresponding patient cannot be identified. Patient data will also be dissociated. Consecutive numbers will be assigned to patients as they are enrolled in the study, and these identification numbers (or codes) will be used in the e-CRF; the full name of the patient will not be included in the e-CRFs. The principal investigator of each center will keep an updated patient identification list containing the name, clinical history number, and identification number (or code) of the patient for the clinical trial.

The study monitor may have access to the patient’s identity and data related to the study monitoring procedures. Anybody with direct access to the data (regulatory authorities, trial monitors and auditors) will take all possible precautions to protect the confidentiality of patient’s identities.

It is the investigator’s responsibility to obtain written informed consent from the study patients. It is the trial monitor’s responsibility to make sure that each patient has given their written consent to allow this direct access.

The investigator shall ensure that the documents provided to the sponsor do not contain the patient’s name or any identifiable data.

### Competing interests {28}

The sponsor will receive from Takeda, the pharmaceutical company that holds marketing authorization for the study molecule (Icatibant), in addition to the study drug (valued at over one million euros), a financial amount to cover the following clinical trial costs: the drafting of the protocol, the administrative procedures to start the clinical trial, the fees of the IRB and the AEMPS, the engagement of a CRO(s) to carry out monitoring, pharmacovigilance, e-CRF preparation, statistical analysis, and the final report. The feasibility of this clinical trial therefore depends on this financial contribution.

An agreement will be drawn up between the study sponsor and Takeda, stating that Takeda will be informed of serious adverse reactions related to icatibant and will be entitled to receive a final clinical trial report, as well as to be informed of any publications (Section 8.2.1). Takeda may provide comments to the sponsor, but it may not impose or interfere with the interpretation of the results or in the final write up of the aforementioned documents.

### Availability of data and materials {29}

The datasets used or analyzed during the study will be available from the corresponding author on reasonable request.

### Provisions for post-trial care {30}

A specific insurance policy has been arranged for ad hoc in case of any harm related to the patient’s participation, if this is not directly caused by the disease under study or by the evolution of any other underlying conditions.

The benefit-risk relationship will not be able to be known till the end of the clinical trial, reason why icatibant will not be given to patients before ending the complete study analysis. However, if the results are promising, this topic could be reconsidered and icatibant could be provided by Takeda at their discretion as long as their assigned physician prescribes it in accordance with usual clinical practice.

### Dissemination plans {31a}

The results will be published in a peer-reviewed journal and presented at international medical conferences.

## Authorship eligibility guidelines and any intended use of professional writers {31b}

The authorship is based on the criteria according to International Committee of Medical Journal Editors

http://www.icmje.org/recommendations/browse/roles-and-responsibilities/defining-the-role-of-authors-and-contributors.html (accessed July 7th 2021).

### Plans to give access to the full protocol, participant-level data, and statistical code {31c}

The protocol is available under EudraCT code: 2020-002166-13 and on clinicaltrials.gov (NCT NCT04978051). No public access to the patient dataset is planned at this time. Dr. Cristian Tebé, Head of the Biostatistics Unit, will be in charge of the dataset and access to this information will be granted on a case-by-case basis and at the request of the interested party.

### Consent for publication {32}

The template for the consent form is available upon request to PM (pierre.malchair@bellvitgehospital.cat) or AO (aoterog@bellvitgehospital.cat).

### Plans for collection, laboratory evaluation, and storage of biological specimens for genetic or molecular analysis in this trial/future use {33}

Blood samples will be systematically collected on baseline visit and on visits 1, 2, 3, and 5, and on visits 4, 6, and 7 according to medical criteria (see Table 3). Blood analysis will include complete blood cells count, D-dimer, sedimentation rate, AST, ALT, bilirubin, creatinine, creatinine clearance, LDH, PT, aPTT, fibrinogen, ferritin, HDL, LDL, triglycerides, troponin, creatine-kinase, c-RP (c-reactive protein), complement factor C4, and C1 esterase inhibitor. These blood samples will be destroyed following the procedure of each center’s clinical practice.

Diagnosis of SARS-CoV-2 will be done by qualitative reverse transcriptase polymerase chain reaction (RT-PCR) from nasopharyngeal smear or through qualitative antigen testing.

## Discussion

The administration of icatibant acetate (Firazyr®) to patients with SARS-CoV-2 infection will allow assessment of its competitive antagonism effect at the bradykinin type 2 receptor on the endothelial cell, acting as a potential anti-inflammatory (which may inhibit edema development) and as a potential inhibition of the SARS-CoV-2 protease.

The rationale for the potential efficacy of icatibant in COVID-19 is based on the inhibition of action of bradykinin on endothelial cells and on the inhibition of the SARS-CoV-2 M protease (a key enzyme in coronavirus replication) [17]. Hence, icatibant may interfere with the inflammatory cascade responsible for tissue damage, as well as support ventilatory function, while endogenous mechanisms and treatments promote recovery in the face of greater impairment in life-threatening conditions [18, 19]. Currently, it is unknown whether this approach (icatibant) could help avoid serious complications from SARS-CoV-2 infection. However, a case-control study in patients with COVID-19 concluded that icatibant had an excellent tolerance and improved the oxygenation of those patients [20]. It is noteworthy that another study protocol of a phase II, single-center, three-armed parallel-group, open-label clinical trial has been recently published [21].

Likewise, there is uncertainty regarding the most appropriate dose and timing of administering icatibant in a SARS-CoV-2 infection setting. In this proof-of-concept clinical trial, we have chosen the dosing regimen of icatibant based on 3 doses per day for 3 consecutive days. Icatibant is characterized by a rapid onset of action, after binding to BK2R, and a rapid relief of clinical symptoms in hereditary angioedema (HAE). It also has a short half-life of approximately 1.4 h (1.48 ± 0.35 h) after a single subcutaneous 30-mg dose [22]. Therefore, the administration of multiple doses at given intervals would be necessary to ensure continuous exposure over several days. It has been estimated that the median time for COVID-19 patients to develop an ARDS is of approximately 8–12 days from the symptom onset (the timeframe for edema development and increase). While it is true that the proposed posology is largely empirical, data from icatibant in HAE has been considered. The option of repeating a dose for up to a maximum of 3 doses in 24 h is permitted if a single 3 mL subcutaneous injection of icatibant 10 mg/mL (a total of 30 mg) is not sufficient to induce complete symptom remission [5]. Albeit no more than 8 icatibant injections per month were administered in previous clinical trials on HAE patients, post-marketing data analysis from the IOS registry, obtained from 557 patients with HAE who were administered icatibant to treat a total of 3025 HAE attacks, shows no evidence of additional risks associated with the administration of 8 doses/month of icatibant, or with repeated administrations at consecutive or smaller timeframes (< 6-h intervals) [6]. Furthermore, repeated subcutaneous administrations of 30 mg icatibant at 6-h intervals in healthy volunteers did not result in substantial drug accumulation [22]. As a safety measure, patients will be monitored throughout the study with special emphasis during the days of icatibant administration and in the 24 h following the end of treatment administration, when clinical status assessment and laboratory analyses will be continuous. Besides, drug safety will be reviewed in depth in the first 10 patients enrolled. If no significant adverse effects are seen in those first 10 patients, patient recruitment will continue. During the treatment period, medical visits (visit 2, visit 3, and visit 4) will be performed for all patients. If any adverse events are found during the clinical trial, they will be described according to severity and association to treatment.

ICAT-COVID study has limitations, such as the open-label design; the novelty of this disease results in a rapidly changing environment regarding SoC guidelines [14], which might influence the ICAT-COVID trial and potentially impose adaptations in the study protocol; and the unpredictability of the COVID-19 pandemic, which leads to uncertainties regarding enrollment rates. The proposed open-label design without a placebo, but with two arms: experimental (SoC-plus-Icatibant) and control (SoC), is considered adequate to obtain the first evidence of this drug as treatment for patients hospitalized for SARS-CoV-2 pneumonia and treated with supplemental oxygen using non-invasive ventilation. The ideal design would be a double-blind, randomized, placebo-controlled clinical trial. However, this design was ruled out for several reasons: (i) The impossibility of on-site placebo manufacturing at the centers’ pharmacies. Since icatibant is a solution for injection in a pre-filled syringe, the placebo should also be a solution for injection in a pre-filled syringe. (ii) The inability to mask pain caused by icatibant at the site of administration. This symptom occurs in practically all patients treated with icatibant and should be recorded in the medical history. These data would reveal which patients receive icatibant and which patients receive placebo. (iii) The manufacture of placebo has been considered in a CRO. However, the time required for the manufacturing process and its costs (which we could not afford) would have made the study unfeasible.

Sample size could be another drawback. At the time of writing the study protocol, data to estimate the sample size are scarce. Consequently, it was decided to conduct a proof-of-concept trial and enroll 120 patients (60 in each study group), a feasible sample size within our means.

We consider that including patients in our clinical trial with SARS-CoV-2 pneumonia and oxygen requirements, but not invasively ventilated (WHO-ordinal scale for clinical assessment 4 and 5), could allow us to evaluate the impact of bradykinin inhibition in the first stages of SARS-CoV-2 infection. If the inflammatory stage of the disease can be reduced, Icatibant could be considered as a potential therapeutic option and further larger trials would be carried out.

## Trial status

The current protocol version is 3.2 (January 2021) and ICAT-COVID study is in the recruitment phase. Recruitment started in April 2021 and is currently ongoing 77 patients have been enrolled at the time of sending this manuscript for publication.

## Supplementary Information

**Additional file 1.** Consent form

## Abbreviations

ACE2: Angiotensin-converting enzyme 2
AE: Adverse event
AEMPS: Spanish Agency for Medicinal Products and Medical Devices
ALT: Alanine aminotransferase
aPTT: Activated partial thromboplastin time
ARDS: Acute respiratory distress syndrome
AST: Aspartate aminotransferase
BK2R: Bradykinin type II receptor
CI: Confidence interval
COVID-19: Coronavirus disease 2019
CRO: Contract Research Organization
c-RP: C-reactive protein
CTCAE: Common Terminology Criteria for Adverse Events
e-CRF: Electronic case report form
EKG: Electrocardiogram
EU: European Union
EudraCT: European Union Drug Regulating Authorities Clinical Trials Database
HAE: Hereditary angioedema
HDL: High-density lipoprotein
ICAT-COVID: ICATIBANT—COVID
ICU: Intensive care unit
IDIBELL: Bellvitge Institute for Biomedical Research
IOS: Icatibant Outcome Survey
IRB: Institutional review board
LDH: Lactate dehydrogenase
LDL: Low-density lipoprotein
LOCF: Last observation carried forward
MedDRA: Medical Dictionary for Regulatory Activities
NEWS-2: National Early Warning Score 2
PaO_2_: Oxygen pressure in arterial blood
PCR: Polymerase chain reaction
PP: Per protocol
PT: Prothrombin time
REDCap: Research Electronic Data Capture
SAE: Serious adverse event
SAR: Serious adverse reaction
SD: Standard deviation
SmPC: Summary of Product Characteristics for the investigational medicinal product
SoC: Standard of care
SpO_2_: Oxygen saturation
SUSARs: Suspected unexpected serious adverse reactions
RT-PCR: Reverse Transcription-polymerase chain reaction
UBiDi: Biostatistics Unit of the IDIBELL
WHO: World Health Organization

## References

[1] Zhu N, Zhang D, Wang W, Li X, Yang B, Song J (2020). China Novel Coronavirus Investigating and Research Team. A novel coronavirus from patients with pneumonia in China, 2019. N Engl J Med.

[2] COVID19, WHO. WHO Coronavirus (COVID-19) Dashboard. https://covid19.who.int/ (accessed February 3^th^, 2022).

[3] Sidiqqi HK, Mehra MR (2020). COVID-19 illness in native and immunosupressed states: a clinical- therapeutic staging proposal. J Heart Lung Transplant.

[4] Cicardi M, Banerji A, Bracho F, Malbrán A, Rosenkranz B, Riedl M, Bork K, Lumry W, Aberer W, Bier H, Bas M, Greve J, Hoffmann TK, Farkas H, Reshef A, Ritchie B, Yang W, Grabbe J, Kivity S, Kreuz W, Levy RJ, Luger T, Obtulowicz K, Schmid-Grendelmeier P, Bull C, Sitkauskiene B, Smith WB, Toubi E, Werner S, Anné S, Björkander J, Bouillet L, Cillari E, Hurewitz D, Jacobson KW, Katelaris CH, Maurer M, Merk H, Bernstein JA, Feighery C, Floccard B, Gleich G, Hébert J, Kaatz M, Keith P, Kirkpatrick CH, Langton D, Martin L, Pichler C, Resnick D, Wombolt D, Romero DSF, Zanichelli A, Arcoleo F, Knolle J, Kravec I, Dong L, Zimmermann J, Rosen K, Fan WT (2010). Icatibant, a new bradykinin-receptor antagonist in hereditary angioedema. N Engl J Med.

[5] Icatibant (Firazyr®). Summary of product characteristics (Ficha técnica, Febrero 2019). https://cima.aemps.es/cima/pdfs/es/ft/08461001/FT_08461001.pdf (accessed July 5^th^, 2021).

[6] Busse PJ, Christiansen SC (2020). Hereditary angioedema. N Engl J Med.

[7] Zanichelli A, Maurer M, Aberer W, Caballero T, Longhurst HJ, Bouillet L, Fabien V, Andresen I, the IOS Study Group (2017). Long-term safety of Icatibant treatment of patients with angioedema in real- world clinical practice. Allergy Eur J Allergy Clin Immunol.

[8] Bygum A, Caballero T, Grumach AS, Longhurst HJ, Bouillet L, Aberer W (2019). Elderly versus younger patients with hereditary angioedema type I/II: patient characteristics and safety analysis from the Icatibant Outcome Survey. Clin Transl Allergy.

[9] Bova M, Guilarte M, Sala-Cunill A, Borrelli P, Rizzelli GM, Zanichelli A (2015). Treatment of ACEI-related angioedema with Icatibant: a case series. Intern Emerg.

[10] Hess R, Wujak L, Hesse C, Sewald K, Jonigk D, Warnecke G, Fieguth HG, Maat S, Maas C, Bonella F, Preissner K, Weiss B, Schaefer L, Kuebler W, Markart P, Wygrecka M (2017). Coagulation factor XII regulates inflammatory responses in human lungs. Thromb Haemost.

[11] Van De Veerdonk FL, Netea MG, van Deuren M, van der Meer JWM, de Mast Q, Brüggemann RJ, et al. Kinins and cytokines in COVID-19: a comprehensive pathophysiological approach. 10.20944/preprints202004.0023.v1.

[12] Tolouian R, Vahed SZ, Ghiyasvand S, Tolouian A, Ardalan M (2020). COVID-19 interactions with angiotensin-converting enzyme 2 (ACE2) and the kinin system; looking at a potential treatment. J Ren Inj Prev.

[13] Yang G, Tan Z, Zhou L, Yang M, Peng L, Liu J, Cai J, Yang R, Han J, Huang Y, He S (2020). Effects of angiotensin II receptor blockers and ACE (angiotensin-converting enzyme) inhibitors on virus infection, inflammatory status, and clinical outcomes in patients with COVID-19 and hypertension: a single-center retrospective study. Hypertension..

[14] COVID-19 guidelines. Pharmacological treatment of SARS-CoV-2 infection. Catalan Health Service. Available in: https://catsalut.gencat.cat/ca/proveidors-professionals/farmacia-medicaments/programa-harmonitzacio-farmacoterapeutica/pautes-harmonitzacio-farmacoterapeutica/covid-19/index.html#googtrans(ca|en) (accessed January 18^th^, 2022)

[15] COVID-19: Treatment Trial Design Master Protocol. World Health Organization 2020. Available in: https://www.who.int/blueprint/priority-diseases/key-action/COVID-19_Treatment_Trial_Design_Master_Protocol_synopsis_Final_18022020.pdf (accessed July 5^th^, 2021).

[16] National Early Warning Score (NEWS) 2, available in: https://www.mdcalc.com/national-early-warning-score-news-2 (accessed July 5^th^, 2021).

[17] Liu X, Wang X (2020). Potential inhibitors against 2019-nCoV coronavirus M protease from clinically approved medicines. J Genet Genomics.

[18] Wang D, Hu B, Hu C, Zhu F, Liu X, Zhang J, Wang B, Xiang H, Cheng Z, Xiong Y, Zhao Y, Li Y, Wang X, Peng Z (2020). Clinical characteristics of 138 hospitalized patients with 2019 novel coronavirus-infected pneumonia in Wuhan, China. JAMA..

[19] Zhou F, Yu T, Du R, Fan G, Liu Y, Liu Z (2020). Clinical course and risk factors for mortality of adult inpatients with COVID-19 in Wuhan, China: a retrospective cohort study. Lancet..

[20] van de Veerdonk FL, Kouijzer IJE, de Nooijer AH, van der Hoeven HG, Maas C, Netea MG (2020). Outcomes associated with use of a kinin B2 receptor antagonist among patients with COVID-19. JAMA Netw Open.

[21] Mansour E, Bueno FF, de Lima-Júnior JC, Palma A, Monfort-Pires M, Bombassaro B, Araujo EP, Bernardes AF, Ulaf RG, Nunes TA, Ribeiro LC, Falcão ALE, Santos TM, Trabasso P, Dertkigil RP, Dertkigil SS, Maia RP, Benaglia T, Moretti ML, Velloso LA (2021). Evaluation of the efficacy and safety of icatibant and C1 esterase/kallikrein inhibitor in severe COVID-19: study protocol for a three-armed randomized controlled trial. Trials..

[22] Leach JK, Spencer K, Mascelli M, McCauley TG (2015). Pharmacokinetics of single and repeat doses of icatibant. Clin Pharmacol Drug Dev.

