# Supplementary material for: A multicenter, open-label, randomized, proof-of-concept phase II clinical trial to assess the efficacy and safety of icatibant in patients infected with SARS-CoV-2 (COVID-19) and admitted to hospital units without invasive mechanical ventilation: study protocol (ICAT-COVID)
Source: Trials. 2022 Apr 12;23:303. doi: 10.1186/s13063-022-06219-7 (PMC9003174; doi:10.1186/s13063-022-06219-7)
Supplement: Supplementary file 1 — Additional file 1. Consent form [file 13063_2022_6219_MOESM1_ESM.pdf]

6.3 Appendix 3: PATIENT INFORMATION SHEET

Study: HUB-MdI-ICAT·COVID-201 – Patient Information Sheet version 3.2 dated 21 January 2021. ICH compliant protocol

Research Project entitled: **A PHASE II, RANDOMISED, OPEN-LABEL, MULTICENTRE, PROOF-OF-CONCEPT CLINICAL TRIAL TO EVALUATE THE EFFICACY AND SAFETY OF ICATIBANT IN PATIENTS INFECTED BY SARS-COV-2 (COVID-19) AND ADMITTED TO HOSPITALISATION UNITS, WITHOUT INVASIVE MECHANICAL VENTILATION, COMPARED TO THE STANDARD OF CARE (ICAT·COVID).**

EudraCT: 2020-002166-13
Coordinating Investigator: Dr. Pierre Malchair.
Internal Medicine Department of Hospital Universitario de Bellvitge.

We are contacting you to inform you about a research study in which you are invited to take part. The study has been approved by a Medicinal Product Research Ethics Committee and by the Spanish Agency for Medicinal Products and Medical Devices, in accordance with applicable law, Royal Decree 1090/2015, of 4 December, and European Regulation 536/2014, of 16 April, regulating clinical trials with medicinal products.

Our aim is that you receive all the accurate information you require to be able to decide whether or not you wish to take part in this study. Therefore, we ask you to please read this information sheet carefully and we will answer any questions you have. You may also consult with anyone you consider appropriate.

Please be aware that your participation in this study is voluntary and you can decide NOT to take part in it. If you decide to participate, you can change your mind and withdraw your consent at any time, without this changing the relationship with your doctor or affecting your medical care in any way.

Introduction: There is currently no known effective treatment for COVID-19. This new disease poses a challenge to the medical community to identify effective treatment options for its treatment and prevention. Currently, there is no approved drug for the treatment of SARS-CoV-2 or coronavirus (COVID-19) infection.

Icatibant acetate (Firazyr®) is an approved and marketed drug. Icatibant is approved for the treatment of acute angioedema attacks in patients diagnosed with hereditary angioedema due to C1 inhibitor deficiency.

There are different reasons, based on studies conducted to determine how this drug works, that make us think that icatibant may be effective for treating SARS-CoV2 coronavirus infection. Icatibant can target COVID-19 through two potential mechanisms: 1) blocking the action of the protein called bradykinin, and 2) inhibiting another protein called coronavirus M-protease.

Bradykinin is a protein we all have. This protein helps blood vessels to dilate and makes it easier for fluid to leak out of the blood vessels causing oedema, just what happens if you get SARS-Cov-2 pneumonia. Icatibant prevents this protein from working and therefore prevents oedema from forming.

The protein called coronavirus M-protease is needed for this virus to replicate and maintain its infective potential. Because of its structure, icatibant could inhibit this protein and therefore disable coronavirus replication.

Our working hypothesis is that treatment with Icatibant, together with the standard care, in patients with pneumonia caused by SARS-CoV-2 (COVID-19) and hospitalised in a non-ventilated ward (as is the case) is more effective than the standard care.

Benefits: Your participation in this study would contribute with data to increase the understanding of the efficacy and safety of anti-Covid treatments, as well as potential predictors of response. However, your participation in this study may mean that you do not obtain any benefit.

Study procedures: It is planned to include a maximum of 120 participants in this study, half will be treated with icatibant for 3 days (3 doses per day, that is, a maximum of 9 doses) together with the standards of care or with the standards of care alone. The procedure for assigning to one of the two treatment groups will be done at random (for example, like tossing a coin), so the chance of falling into one group or another is 50%.

The standards of care include those basic treatments required for the symptoms of each patient, such as hydration with fluids, drugs to reduce fever, relieve nausea or calm pain, bronchodilators in case of bronchospasm, anti-inflammatory and antiviral treatment, in addition to adjustment of regular medication. In addition, Hospital Universitario de Bellvitge has an intervention protocol for the care and treatment of COVID-19 infected patients.

If you decide to participate, a first visit will be performed to verify that you can meet the criteria to participate in the study. Once you are included in the study, you will be assigned to one of the treatments discussed above.

Demographic and medication information will be collected. You will not undergo any additional tests, that is, the tests involved for participating in the study are the same as those you would undergo in the management of your disease.

Once you are discharged from the hospital, during the different study-related visits, you will be asked to report any adverse events you experience. In addition, you should not modify the medication you are taking or take other medicines or "medicinal plants" without first consulting with the study doctor.

Possible risks, discomforts and side effects related to icatibant treatment. As it is a marketed drug approved by the competent health authorities, there is information available worldwide on side effects. This drug been used for more than 10 years for the treatment of acute angioedema attack in patients diagnosed with hereditary angioedema due to C1 inhibitor deficiency, though it has not been used in patients with COVID-19. Please talk to your study doctor to obtain a complete list of the side effects reported with these drugs and in any case you will be given the package leaflet for the drugs you are treated with. The most common side effects are injection site reactions (irritation, swelling, pain, itching, redness, burning). These reactions are usually mild to moderate, transient, and improve on their own. In addition, some patients may experience headache, dizziness, increased body temperature and increased liver enzymes known as transaminases.

Any new information regarding the treatments used in the study that could affect your decision to continue in the study will be reported by the doctor as soon as possible and, if necessary, a new consent will be signed.

If you decide to participate, it is proposed to closely monitor any adverse effect described for icatibant to ensure your safety.

If you are a woman participating in the study and a pregnancy occurs during your participation in the study (up to day 28 from hospital discharge), you should inform your doctor immediately to receive adequate medical care, to assess if you need to be withdrawn from the trial and to monitor your pregnancy, for your and your fetus's safety. If you are a male study participant, and your partner becomes pregnant during the study period (up to day 28 from hospital discharge), you should inform your doctor immediately so that your partner receives adequate medical care, your doctor may ask you for information about the course of the pregnancy and its outcome, or, if necessary, through a specific informed consent form for your pregnant partner.

This study complies with current legislation (Royal Decree 1090/2015). A specific insurance policy has been taken out for this study in the event of any harm to your health or injuries that could occur in relation to your participation in the study, provided they are not a consequence of the disease being studied or of the progression of your underlying disease. If you would like more information regarding this section, please consult the principal investigator of this study.

Please be informed that your participation in this clinical trial could change the general and particular terms and conditions (coverage) of your insurance policies (life, health, accident, etc.). Therefore, we recommend that you contact your insurance company to determine whether your participation in this study will affect your current insurance policy.

The study sponsor is responsible for managing its funding. You will not have to pay for the drugs or specific tests that may result from your participation in the study. Your participation in this study will involve no additional cost for you.

Other relevant information:

You should know that you may be excluded from the study if the sponsor or the study investigators consider it appropriate, whether for reasons of safety, adverse events caused by the study drug, or because they consider that you are not complying with the established procedures. In all these cases, you will be given an adequate explanation about why you have been withdrawn from the study.

In the event of early termination of the trial by the sponsor, the participants will be duly informed of the reasons.

Personal data protection:

Both the sponsor and the site are respectively responsible for processing your data and undertake to comply with Regulation (EU) 2016/679 of the European Parliament and of the Council of 27 April 2016 on Data Protection (GDPR), as well as all other applicable laws and regulations (Organic Law on Personal Data Protection and Guarantee of Digital Rights 3/2018 of 05 December).

Both the site and the sponsor are responsible for processing of your data and undertake to comply with current data protection regulations. The data collected for the study will be identified with a code, so that it does not include information that could identify you, and only your study doctor/co-investigators will be able to link such data to you and to your medical record. Therefore, your identity will not be disclosed to any other person except the health authorities, when required by law or in the event of a medical emergency.

Access to your personal information will be restricted to the research ethics committees, representatives of the health authorities for inspection purposes (Spanish Agency of Medicinal Products and Medical Devices, foreign health authorities) and staff authorised by the sponsor (monitors, auditors) when required to verify your personal data, clinical study procedures and compliance with good clinical practice guidelines, but maintaining confidentiality of the information at all times.

The data will be entered to a research file held by the institution and processed within the framework of your participation in this study. The data from this study may be used for future research. The sponsor will adopt the necessary measures to guarantee the protection of your privacy and to not allow your data to appear in any other databases that could disclose your identity.

According to data protection legislation, you may exercise your right to access, modify, oppose and cancel your data, by contacting your study doctor. In addition, you may also limit processing of data that are incorrect, request a copy or restrict transfer to a third party (portability) of the data you have provided for the study. To exercise your rights, contact the principal investigator of the study or the Data Protection Officer of the institution (ICS), or otherwise contact the sponsor's data protection unit (email: dataprotection@idibell.cat).

If you decide to withdraw your consent to participate in this study, in order to guarantee the validity of the research and to comply with the legal duties and medicinal product authorisation requirements, the data that has already been collected cannot be deleted. In addition, this study will consult your electronic medical record to verify safety follow-up data one month after your discharge. You also have the right to contact the Data Protection Agency if you are not satisfied.

The investigator and the sponsor are required to keep the data collected for the study for at least 25 years after its completion. Subsequently, your personal information will only be kept by the healthcare site and by the sponsor for other scientific research purposes if you gave your consent for this, and if it is permitted by the law and applicable

ethical requirements.
If we transfer your coded data outside the European Union to entities related to our group, service providers or scientific researchers working with us, the participant's data will be protected with safeguards such as contracts or other mechanisms by the data protection authorities. If the participant would like to know more about this, he/she can contact the Sponsor's Data Protection Officer (Eroni Volavola; dpo_italfarmaco@itfsp.com).

If you need more information about this study, you can contact the **investigator in charge**, Dr.
______________________________Department of______________of Hospital__________________, on the floor.
Contact phone number:_________________________.

WHAT TREATMENT WILL I RECEIVE WHENTHE CLINICAL TRIAL ENDS?
When your participation ends, you will receive the best treatment available that your doctor considers most appropriate for your disease. Therefore, neither the investigator nor the sponsor accepts any commitment to maintain this treatment outside of this study.

INFORMED CONSENT FORM

Study title: "**A PHASE II, PROOF-OF-CONCEPT, RANDOMISED, OPEN-LABEL, MULTICENTRE CLINICAL TRIAL TO EVALUATE THE EFFICACY AND SAFETY OF ICATIBANT IN PATIENTS INFECTED WITH SARS-COV-2 (COVID-19) AND ADMITTED TO HOSPITALISATION UNITS, WITHOUT INVASIVE MECHANICAL VENTILATION, COMPARED TO THE STANDARD OF CARE (ICAT·COVID).**"

Study code: HUB-MdI-ICAT·COVID-201

I, (full name)________________________________.

I have read the information sheet about the study which has been given to me.
I have been able to ask questions about the study.
I have received enough information about the study.
I have talked with (full name of the investigator): ______________________________

I also understand that my participation is voluntary and that I can withdraw from the study:

1. Whenever I wish.
2. Without having to give explanations.
3. Without this decision affecting my medical care.

I will be given a signed and dated copy of this informed consent form. I freely consent to

participate in the study.

____________________	____________________
Signature of the participant	Signature of investigator
Date:____/___/____	Date:____/___/____

When IC is obtained in persons with modified capacity to give their IC

____________________	____________________
Signature of the legal representative representative, family member or related person	Signature of legal
Date:____/___/____	Date:____/___/____
